# Comparing the Ground Reaction Forces, Toe Clearances, and Stride Lengths of Young and Older Adults Using a Novel Shoe Sensor System

**DOI:** 10.3390/s24216871

**Published:** 2024-10-26

**Authors:** Hide Matsumoto, Masaki Tomosada, Toshiaki Nishi, Yoshihiro Sasaki, Ryota Sakurai, Takeshi Yamaguchi

**Affiliations:** 1 Graduate School of Engineering, Tohoku University, Sendai 980-8579, Japan; hide.matsumoto.r4@dc.tohoku.ac.jp (H.M.); masaki.tomosada.p2@dc.tohoku.ac.jp (M.T.); toshiaki.nishi.b3@tohoku.ac.jp (T.N.); 2 Research Institute for Electromagnetic Materials, Tomiya 981-3341, Japan; sasaki@denjiken.ne.jp; 3 Tokyo Metropolitan Institute for Geriatrics and Gerontology, Itabashi 173-0015, Japan; r_sakurai@hotmail.co.jp; 4 Graduate School of Biomedical Engineering, Tohoku University, Sendai 980-8579, Japan

**Keywords:** shoe sensor system, aging, gait, ground reaction force, stride length, toe clearance

## Abstract

In this study, we developed a lightweight shoe sensor system equipped with four high-capacity, compact triaxial force sensors and an inertial measurement unit. Remarkably, this system enabled measurements of localized three-directional ground reaction forces (GRFs) at each sensor position (heel, first and fifth metatarsal heads, and toe) and estimations of stride length and toe clearance during walking. Compared to conventional optical motion analysis systems, the developed sensor system provided relatively accurate results for stride length and minimum toe clearance. To test the performance of the system, 15 older and 8 young adults were instructed to walk along a straight line while wearing the system. The results reveal that compared to the young adults, older adults exhibited lower localized GRF contributions from the heel and greater localized GRF contribution from the toe and fifth metatarsal locations. Furthermore, the older adults exhibited greater variability in their stride length and smaller toe clearance with greater variability compared to the young adults. These results underscore the effectiveness of the proposed gait analysis system in distinguishing the gait characteristics of young and older adults, potentially replacing traditional motion capture systems and force plates in gait analysis.

## 1. Introduction

With the intensification of the demographic shift toward an aging population, falling incidents among older adults are becoming more frequent [1,2]. Aging adversely impacts gait performance, leading to reduced gait speed and stride length, along with increased variability in these parameters and foot clearance [3,4,5,6]. Studies have consistently demonstrated that such increased variability in the parameters above is associated with a heightened risk of falls among the elderly [3,7,8,9]. Furthermore, these variations in gait parameters are associated with alterations in kinetic parameters, such as ground reaction force (GRF), which impact movement dynamics [9,10,11]. Hence, measuring and monitoring these kinetic and kinematic parameters during walking can help identify older adults who are at a high risk of falling.

In the context of gait analysis, monitoring key kinetic and kinematic parameters, such as GRF [12,13,14], walking velocity [15,16,17,18], step length [15,19,20], step width [19,20], and foot clearance [3,21], is essential. Typically, this monitoring relies on three-dimensional motion analysis systems, comprising force plates and motion capture systems. However, these systems present certain limitations. For instance, they are expensive, require long periods for setup, and demand extensive, dedicated spaces, rendering them impractical in numerous clinical and everyday settings [22,23]. Furthermore, they are incapable of localized GRF measurements, which are vital for assessing the contact and interaction dynamics of a foot and floor during walking.

Conversely, shoe-based gait analysis systems offer enhanced flexibility by eliminating location constraints. These systems typically rely on insole-type pressure sensors or triaxial force sensors for GRF measurements. [24]. Notably, insole-type pressure sensors can measure vertical plantar pressures, thereby enabling accurate estimation of the vertical GRF component [25,26,27]. However, the estimations of the anterior–posterior and mediolateral GRF components from planter pressure data necessitate mathematical models, such as machine learning algorithms [28,29,30,31,32]. Thus, GRF measurements based on insole-type pressure sensors exhibit suboptimal estimation accuracies and poor robustness when applied to diverse walking models and real-world walking conditions. Furthermore, despite their cost-effectiveness, limitations in terms of sensor longevity and sensitivity to boundary conditions in shoes hinder long-term monitoring [24]. In contrast, direct GRF measurements using triaxial force sensors offer greater measurement accuracy than GRF measurements based on insole sensors [24]. However, large triaxial force sensors can interfere with the natural gait [33,34,35], whereas small triaxial sensors often lack the capacity to measure large forces at the heel [36,37,38]. In light of this, we recently developed a shoe-based sensor system with compact, high-capacity triaxial force sensors capable of measuring GRFs without interfering with the natural gait [39]. This system can provide three-dimensional, localized GRF distributions across plantar surfaces during walking.

Previous studies have developed shoe-based gait analysis systems equipped with an inertial measurement unit (IMU) to assess kinematic parameters, such as stride length and foot clearance [40,41,42,43,44,45,46,47,48]. In these studies, stride length is calculated through the second order integration of acceleration signals measured by a single IMU attached to the shoe. This approach models the drift associated with integration as a piecewise linear function, assuming zero velocity at ground contact [49]. Adopting the same method, Benoussaad et al. [42] estimated the foot clearance and achieved a root mean square error (RMSE) of 0.0074 m, surpassing the accuracy required for clinical practice in normal walking. In addition, the precision of the minimum foot clearance estimation has been further improved through machine learning and other advanced techniques [43].

Although shoe-based gait analysis systems have been developed to measure or estimate GRFs, stride length, and foot clearance, most of these systems assess GRFs and kinematic variables independently or integrate uniaxial pressure sensors and IMUs [50,51]. Consequently, shoe-based gait analysis systems capable of simultaneously measuring or estimating three-directional GRFs and kinematic parameters using triaxial force sensors and IMUs remain to be developed. Since the three-axis force sensor tilts to contact the floor surface in response to foot motion, the foot posture angle must be used to correct the force sensor output. By using an IMU in addition to the force sensors, the angle information of the IMU can be used to correct the three-axis force sensor output.

The primary aim of this study was to develop and validate a lightweight shoe sensor system. This system included four compact, high-capacity triaxial force sensors and one IMU, attached to the shoe’s tip closest to the floor during the swing phase. The distance between the shoe tip and the floor was defined as the toe clearance. To further examine the measurement validity, we assessed the gait characteristics of young and older adults using our system as a second objective. Specifically, this was conducted to verify whether the device can confirm previously reported age-related changes and to investigate differences in triaxial local GRF distribution between the two groups, which have never been revealed. To achieve these objectives, two studies were conducted: Study 1 (S1) focused on developing the shoe sensor system, estimating the stride length and toe clearance using data obtained from this system, and comparing these data with values extracted from an optical motion capture system. Meanwhile, Study 2 (S2) focused on measuring and estimating the localized triaxial GRFs, stride lengths, and minimum toe clearances and their variability among young and older adults during straight walking using the shoe sensor shoe system. 

## 2. Methods

### 2.1. Study 1 (S1)

In S1, the shoe sensor system was developed, and the stride length and minimum toe clearance was compared with that of an optical motion capture system.

#### 2.1.1. Developing the Shoe Sensor System

**Figure 1:**
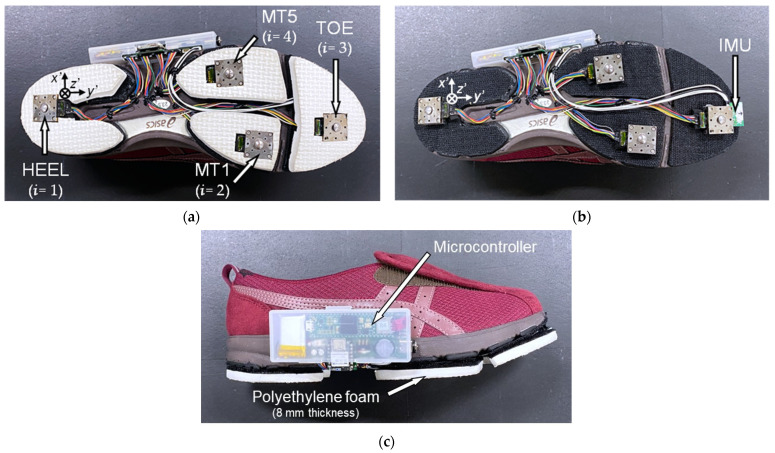
Shoe sensor system. Here, *x*′, *y*′, and *z*′ denote the local coordinates of each sensor system. (**a**) Installation location of the four triaxial force sensors, (**b**) installation location of the inertial measurement unit (IMU, depicted without the polyethylene foam outsole), and (**c**) side view of the shoe sensor system. MT1 and MT5 represent the first and fifth metatarsal heads, respectively.

Figure 1 illustrates the shoe sensor system developed in this study. To assemble this system, an 8 mm thick polyethylene foam outsole was attached to the sole of each walking shoe from a pair (LifeWalker Women’s FLC307, sizes: 23.5 cm and 25.0 cm; ASICS, Kobe, Japan). Subsequently, a triaxial force sensor with a Cr–N thin-film (Research Institute for Electromagnetic Materials, Tomiya, Japan; dimensions: 20 mm × 20 mm × 7.5 mm; mass: 18 g) [52] was affixed to a partially cut-out portion of the sole. This sensor comprised a 20 mm square stainless-steel housing and a force-sensing contactor (lever). In total, eight sensors—four in each shoe—were used to simultaneously measure forces in three directions at various locations. Each force sensor was individually calibrated before installation on the shoes. The surface of each sensor housing was adjusted to align with the polyethylene foam surface, with the protruding lever (3.0 mm in height) receiving the applied force. The sensors were enclosed within 1 mm thick nitrile rubber to minimize abrasion between the sensor contactor and ground. In this arrangement, the sensors protruded approximately 4 mm beyond the shoe surface. Given that the sole of the shoe is not a rigid surface, parts of the sole other than the force sensors make contact with the floor owing to deformation. Therefore, not all GRFs are exerted on the sensors. An IMU (9-DOF Absolute Orientation IMU Fusion Breakout-BNO055; Adafruit, NY, USA; dimensions: 18 mm × 11 mm × 4 mm; mass: 0.6 g) was then mounted at the toe of the shoe at a distance of 3 mm above the ground using adhesive, as illustrated in Figure 1b. The force sensors and IMU were connected to a microcontroller (Teensy 3.6, SparkFun, Electronics®, Niwot, CO, USA). Additionally, the microcontroller board and battery were enclosed in a case, which was attached to the side of the shoe. The microcontroller was wired to a trigger and activated by pressing a switch, which initiated data recording on an SD memory card. The force sensors and IMU had sampling frequencies of approximately 400 Hz and 70 Hz, respectively. The total weight of each shoe, including the sensors, battery, and board, was 309 g. As depicted in Figure 1, in the local coordinate system of each force sensor and the IMU, the *x*′, *y*′, and *z*′ directions represent the foot width, foot length, and vertical direction of the shoe, respectively. Furthermore, the three-directional forces measured by the triaxial sensors are denoted as $F_{x^{\prime }i}$, $F_{y^{\prime }i}$, and $F_{z^{\prime }i}$, respectively, with *i* denoting the position of the sensor, where *i* = 1, 2, 3, and 4 denote the heel, first metatarsal, fifth metatarsal, and toe, respectively.

#### 2.1.2. Data Processing Framework of the Gait Analysis System Using Shoe Sensor System

**Figure 2:**
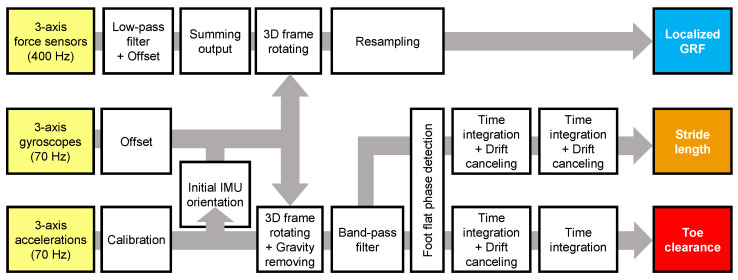
Flowchart of the algorithm used for analyzing localized ground reaction forces (GRFs), stride length, and toe clearance based on data extracted from the shoe sensor system.

The methods used for analyzing the localized GRFs, stride length, and toe clearance using force sensor and IMU data are outlined below, while the flow chart of the algorithm for data analysis is illustrated in Figure 2. Notably, all the subsequent analyses were conducted using MATLAB ver. 9.14.0.2239454 (Mathworks, Natick, MA, USA).

First, the acceleration data were calibrated. For this, the IMU was positioned in 20 random stationary postures, and the calibration coefficients of acceleration and bias values were determined using a Newton iterative optimization algorithm. This process ensured that the composite acceleration in all three directions was equal to the norm of gravitational acceleration (=9.81 m/s^2^) [42,53].

Next, the initial angles $\theta_{0}$ around the *x*′-axis and $\varphi_{0}$ around the *y*′-axis in the initial stationary position of the IMU were estimated as follows using the calibrated acceleration data (*a_x_*_0_, *a_y_*_0_, *a_z_*_0_) [42,54].

(1) $$\theta_{0}=-\arctan\left(\frac{a_{z0}}{a_{x0}}\right).$$

(2) $$\varphi_{0}=\arctan\left(\frac{a_{x0}}{\sqrt{{a_{y0}}^{2}+{a_{z0}}^{2}}}\right).$$

The initial angle $\psi_{0}$ around the z′-axis in the stationary position of the IMU was set to 0°. During walking, the IMU angles θ, φ, and ψ around each axis were obtained by sequentially adding the Euler angles, calculated based on the quaternion output derived from the IMU to the initial angles $\theta_{0}$, $\varphi_{0}$, and $\psi_{0}$, respectively [42].

To calculate the localized GRFs, the time-series data of $F_{x^{\prime }i}$, $F_{y^{\prime }i}$, and $F_{z^{\prime }i}$ (*i* = 1–4) recorded during the stance phase were analyzed. A fourth-order Butterworth low-pass filter with a cutoff frequency of 50 Hz was applied to these data to eliminate noise, and then the offsets were removed [39]. Next, using angles θ and φ, the localized GRF data ($F_{x^{\prime }i}{,F}_{y^{\prime }i}{,F}_{z^{\prime }i}$) were transformed into a horizontal and vertical coordinate system ($F_{xi}{,F}_{yi}{,F}_{zi}$) on the ground, as follows.

(3) $$\left[\begin{array}{c} F_{xi}\\ F_{yi}\\ F_{zi} \end{array}\right]=\left[\begin{array}{ccc} \cos\varphi & 0 & \sin\varphi \\ 0 & 1 & 0\\ -\sin\varphi & 0 & \cos\varphi \end{array}\right]\left[\begin{array}{ccc} 1 & 0 & 0\\ 0 & \cos\theta & -\sin\theta \\ 0 & \sin\theta & \cos\theta \end{array}\right]\left[\begin{array}{c} F_{x^{\prime }i}\\ F_{y^{\prime }i}\\ F_{z^{\prime }i} \end{array}\right]$$

**Figure 3:**
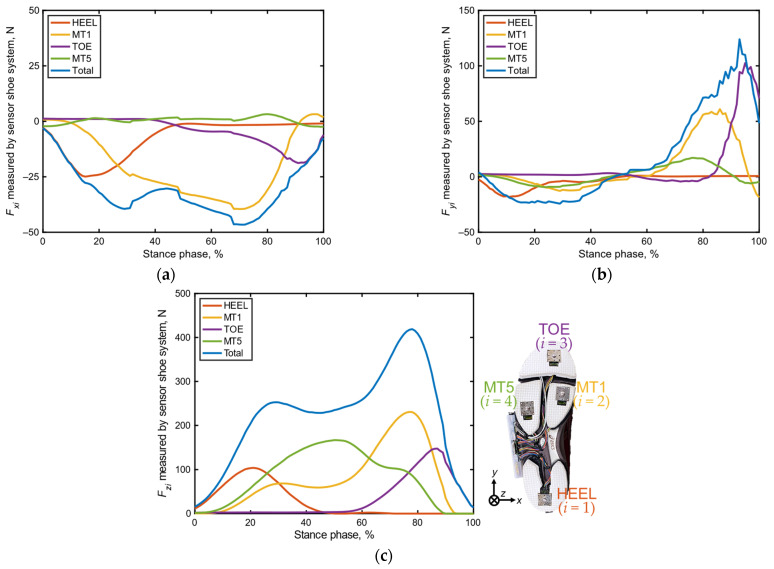
Example of time-series localized GRF ($F_{xi}{,F}_{yi}{,F}_{zi}$ [*i* = 1–4]) and total GRF $\sum_{i=1}^{i=4}f_{zi}$ data recorded by the shoe sensor system during a stance phase. (**a**) Forces in the *x* direction, (**b**) *y* direction, and (**c**) *z* direction. The blue lines represent the total of the four localized GRFs.

In the shoe sensor system, the stance phase was determined by monitoring the total value of $F_{zi}$ (*i* = 1–4). In particular, the beginning of the stance phase was marked by the instant at which $\sum_{i=1}^{i=4}F_{zi}$ exceeded 15 N, while its end was signified by the instant at which $\sum_{i=1}^{i=4}F_{zi}$ fell below 15 N, based on previous research [39]. Notably, the data in each stance phase were regrouped into 101 datasets, with 0% representing heel contact and 100% denoting toe off, to facilitate a comparison of time-series changes during each trial. Figure 3 presents an example of the time-series changes observed in the localized GRFs during a stance phase, as recorded by the shoe sensor system.

To estimate stride length, the acceleration outputs (*a_x′_*, *a_y′_*, *a_z′_*) from the IMU were converted into the global coordinate system (*a_x_*, *a_y_*, *a_z_*) as follows.

(4) $$\left[\begin{array}{c} a_{x}\\ a_{y}\\ a_{z} \end{array}\right]=\left[\begin{array}{ccc} \cos\psi & -\sin\psi & 0\\ \sin\psi & \cos\psi & 0\\ 0 & 0 & 1 \end{array}\right]\left[\begin{array}{ccc} \cos\varphi & 0 & \sin\varphi \\ 0 & 1 & 0\\ -\sin\varphi & 0 & \cos\varphi \end{array}\right]\left[\begin{array}{ccc} 1 & 0 & 0\\ 0 & \cos\theta & -\sin\theta \\ 0 & \sin\theta & \cos\theta \end{array}\right]\left[\begin{array}{c} a_{x^{\prime }}\\ a_{y^{\prime }}\\ a_{z^{\prime }} \end{array}\right]$$

**Figure 4:**
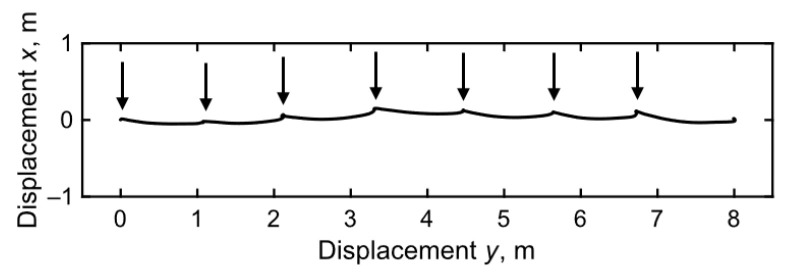
Example of the horizontal trajectory of the IMU position (x, y) at the toe part, as recorded by the shoe sensor system. The arrow indicates the start of the leg swing.

The acceleration data were first processed to eliminate the contribution of gravitational acceleration and then filtered using a fourth-order Butterworth band-pass filter with cutoff frequencies of 1.0 and 40 Hz, which minimized estimation errors. Following this, time integration was performed on the horizontal accelerations *a_x_* and *a_y_* to compute the velocity of the IMU along the horizontal directions. To suppress the drift originating from the integration of acceleration data, a flat-foot phase detection algorithm was implemented [42]. Notably, this algorithm segments the gait into individual strides, preventing the accumulation of drift errors between strides. However, at the end of each stride, a local drift may occur, thereby manifesting as a discrepancy between the integrated data for the flat-foot phase and the expected theoretical data. To address this, drift cancellation was applied to the vertical foot velocity of each stride based on the error accumulated at the end of each stride and the zero vertical velocity assumption [55]. The horizontal position was subsequently calculated by integrating the velocity data over time. Thereafter, the stride length was computed as the difference between the horizontal IMU positions recorded at the start of one swing phase and at the start of the next swing phase, as illustrated in Figure 4.

**Figure 5:**
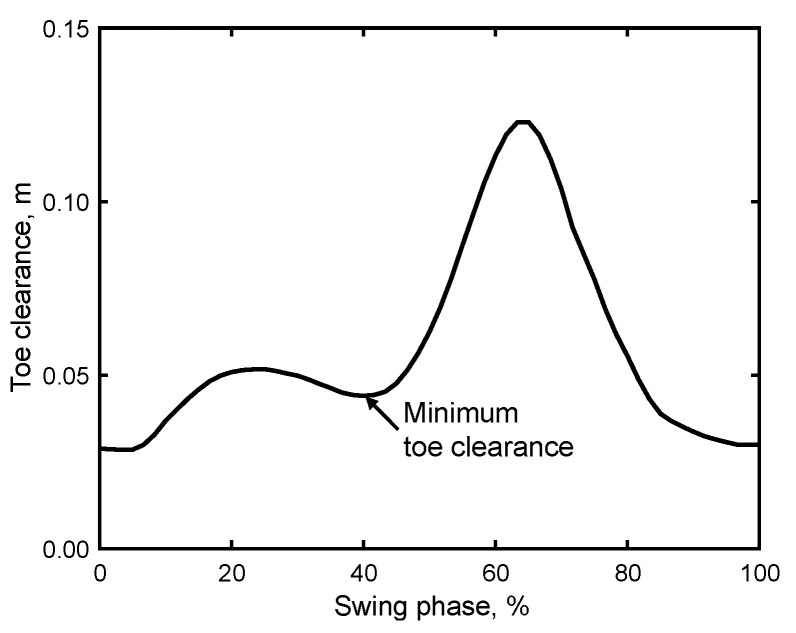
Example of time-series toe clearance data recorded by the shoe sensor system during a swing phase.

The estimation of toe clearance also followed a similar approach using the flat-foot phase detection algorithm [42]. In particular, time integration was performed on the vertical acceleration $a_{z}$ after eliminating gravitational effects. In this case, a zero vertical velocity assumption was applied to correct integration errors. This assumption was based on the zero vertical velocity recorded during the flat-foot phase. The vertical IMU displacement was then computed by integrating the velocity and applying the same drift cancelation on this vertical foot displacement, assuming zero foot displacement at the end of the stride (flat-foot phase). Furthermore, although the IMU was mounted at a height of 0.03 m above the ground, the toe clearance was computed as the vertical IMU travel distance plus the initial height. The minimum toe clearance was then determined by identifying the lowest value in the middle of the swing phase [3,6,21], as illustrated in Figure 5.

#### 2.1.3. Verification Test for the Estimation Accuracy of Stride Length and Minimum Toe Clearance

Fourteen young adults (seven females; age: 22.6 ± 1.6 years; height: 1.66 ± 0.054 m; and body mass: 54.5 ± 5.7 kg) participated in this walking experiment. The experimental protocol was approved in advance by the Ethics Committee for Human Subjects Research, Graduate School of Engineering, Tohoku University (20A-5). Written informed consent was obtained from each participant before the experiments.

During the experiments, the participants were instructed to walk at their normal pace along a 5 m straight path equipped with two force plates at the center. Each participant was instructed to complete 20 walking cycles in the same direction, resulting in 20 strides (10 strides per side) for analysis. Two infrared reflective markers were attached to both sides of the IMU installed on the sole at the toe of the shoe, ensuring that the midpoint between the markers aligned with the center of the IMU. Notably, the sampling frequency of the motion capture system (OptiTrack, Acuity Inc., Reston, VA, USA) was 200 Hz. Subsequently, the gait parameters recorded by the shoe sensor system were compared with those estimated by the motion capture system. The beginning of the stance phase on the force plate was marked by the instant when the vertical GRF exceeded 50 N, while its end was marked by the instant when the vertical GRF fell below 50 N, based on previous research [39,56,57]. The stride length of the motion capture system was calculated as the horizontal difference between the midpoints of the two markers recorded at the start and end of the stance phase. Furthermore, the toe clearance was calculated as the height of the midpoint of the two markers.

The accuracy of the calculations above was assessed using the Pearson product-moment correlation coefficient (*r*) and root mean squared error (RMSE) between the estimated and measured values for each participant [39]. The results were also visualized using Bland–Altman plots, which display the mean values of the estimated and measured data on the *x*-axis and the differences between the estimated and measured data on the *y*-axis. The plots also include a dashed line at ±1.96*σ*, where *σ* denotes the standard deviation of the difference.

### 2.2. Study 2 (S2)

In S2, we used the shoe sensor system to measure and estimate the localized GRFs, stride lengths, and minimum toe clearances of both young and older adults during straight walking. The objective of this analysis was to determine whether the shoe sensor system could highlight differences in gait characteristics between these age groups.

In total, eight young adults (three females) and 15 older adults (all females) participated in this walking experiment. The mean ± standard deviation values of the age, height, and body mass of the participants were 31.1 ± 9.8 years, 1.67 ± 0.066 m, and 60.2 ± 16.6 kg for the young adults and 75.3 ± 4.5 years, 1.54 ± 0.054 m, and 54.6 ± 7.9 kg for the older adults, respectively. The experimental protocol was approved by the Ethics Committee for Human Subjects Research, Tokyo Metropolitan Geriatric Hospital and Institute of Gerontology (R21-20). Written informed consent was obtained from each participant before the experiment.

During the experiment, the participants were instructed to walk at their normal pace along a 10 m straight walking path lined with vinyl composition tiles, starting from a stationary standing position. In total, 10 strides per participant—five strides on each side—were included in the analysis, excluding the initial and final strides of the walk.

Next, we calculated the mean values of the total GRF $(\sum_{i=1}^{i=4}f_{xi},\;\sum_{i=1}^{i=4}f_{yi},$ and $\sum_{i=1}^{i=4}f_{zi}$) normalized by the participants’ body mass for each 10% segment of the stance phase. Additionally, we calculated the mean values and coefficients of variation (CVs) for the stride length and minimum toe clearance data of each participant. A statistically significant difference in height was observed between the young and older participants (unpaired *t*-test, *p* < 0.001), while a positive correlation was observed between height and kinetic parameters (*r* > 0.4). Consequently, we normalized the kinetic parameters by the participants’ heights.

Next, we performed unpaired *t*-tests to compare the group mean values of the total GRF, percentage contributions of the localized GRFs at each stance phase between the two age groups. We also performed unpaired *t*-tests to compare the group mean and CV values of the stride length and minimum toe clearance to identify differences between the age groups. The significance level for this analysis was set to *p* = 0.05. Furthermore, Cohen’s *d*, an effect size, was used to evaluate the differences in the variables above across the considered age groups [58].

## 3. Results

### 3.1. Accuracy Verification of Stride Length and Minimum Toe Clearance (S1)

**Figure 6:**
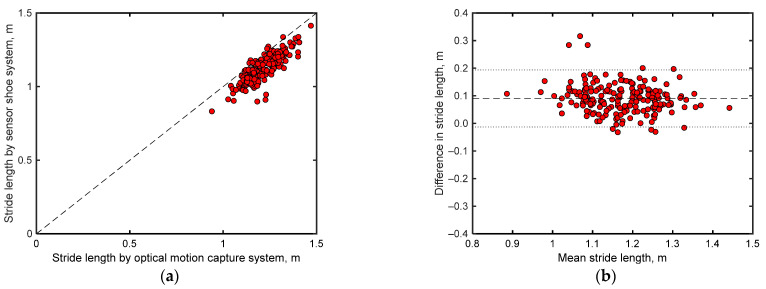
(**a**) Comparisons between mean stride lengths obtained by the motion capture and shoe sensor systems. (**b**) Bland–Altman plots comparing the stride lengths obtained by both systems. Here, the *x*-axes represent the arithmetic means of the readings of both systems, whereas the *y*-axes depict the differences between the readings of both systems. The dashed line in the middle indicates the arithmetic mean of the differences, whereas the lines above and below this line mark the range of ±1.96σ (σ = standard deviation), encompassing 95% of the differences.

Figure 6a compares the mean stride lengths of each participant obtained from the motion capture system and shoe sensor system. Notably, the stride lengths estimated by both systems demonstrate relatively good agreement (*r* = 0.840 with *p* < 0.001 and RMSE = 0.10 m). However, as shown in the Bland–Altman plots (Figure 6b), the stride lengths obtained by both systems exhibit a fixed error of 0.09 m, with most differences in their readings lying within the range of 1.96σ.

**Figure 7:**
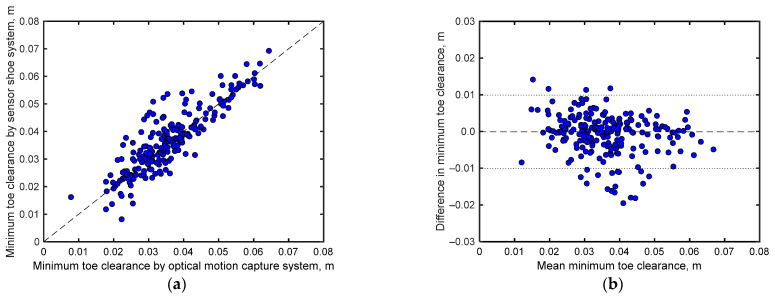
(**a**) Comparison between mean minimum toe clearance obtained from the motion capture and shoe-based sensor systems. (**b**) Bland–Altman plots comparing the minimum toe clearance measurements of both systems. Here, the *x*-axes represent the arithmetic means of the readings from both systems, whereas the *y*-axes depict the differences between these readings. The dashed line in the center indicates the arithmetic mean of the differences, whereas the lines above and below this line mark the range of ±1.96σ (σ = standard deviation), encompassing 95% of the differences.

Figure 7a compares the minimum toe clearance data of each participant recorded by the motion capture and shoe-based sensor systems. Notably, the minimum toe clearances estimated by both systems demonstrate a strong positive correlation (*r* = 0.870 with *p* < 0.001) with an RMSE of 0.0056 m, indicating good agreement. The Bland–Altman plots for the minimum toe clearances (Figure 7b) recorded by both systems exhibit a small fixed error of less than 0.001 m, with most differences in their readings lying within the range of 1.96σ.

### 3.2. Comparison of Gait Parameters Between Young and Older Adults (S2)

#### 3.2.1. Total Ground Reaction Forces (GRF)

**Figure 8:**
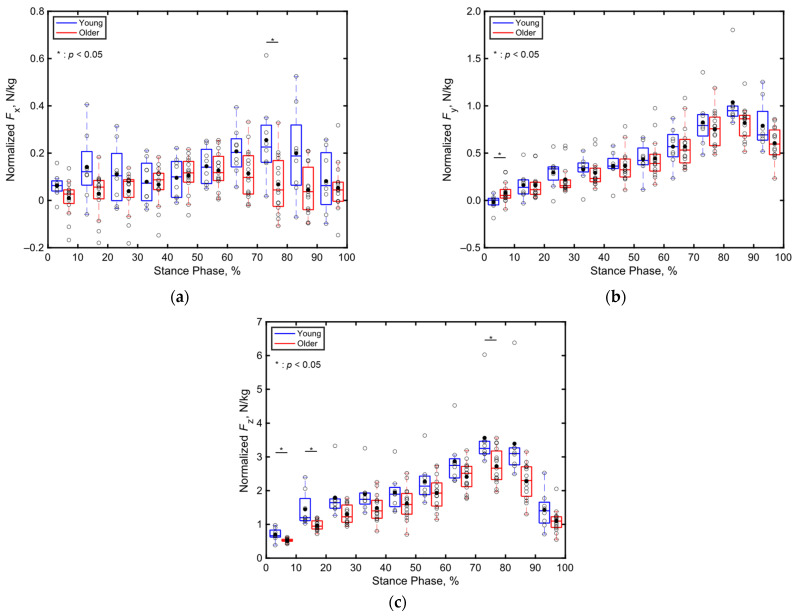
Boxplots of the total triaxial GRF of each participant normalized by their body mass during the stance phase, divided into 10% increments along (**a**) the *x*, (**b**) *y*, and (**c**) *z* directions. Unfilled markers represent the average values for each participant, while black markers denote the mean values for each age group. * indicates *p* < 0.05.

Figure 8 presents boxplots depicting the total GRF normalized by the body mass of each participant from the young and older adult groups across every 10% segment of the stance phase along (a) the *x*, (b) *y*, and (c) *z* directions. Notably, significant differences are apparent in the total GRFs of the age groups along the *x* direction during 71–80% of the stance phase, in the *y* direction during 1–10% of the stance phase, and in the *z* direction during 1–20% and 81–90% of the stance phase (*p* < 0.05, Cohen’s d > 0.8). However, no significant differences are observed in other parts of the stance phase along the *x*, *y*, and *z* directions.

#### 3.2.2. Percentage Contributions of Localized GRFs

**Figure 9:**
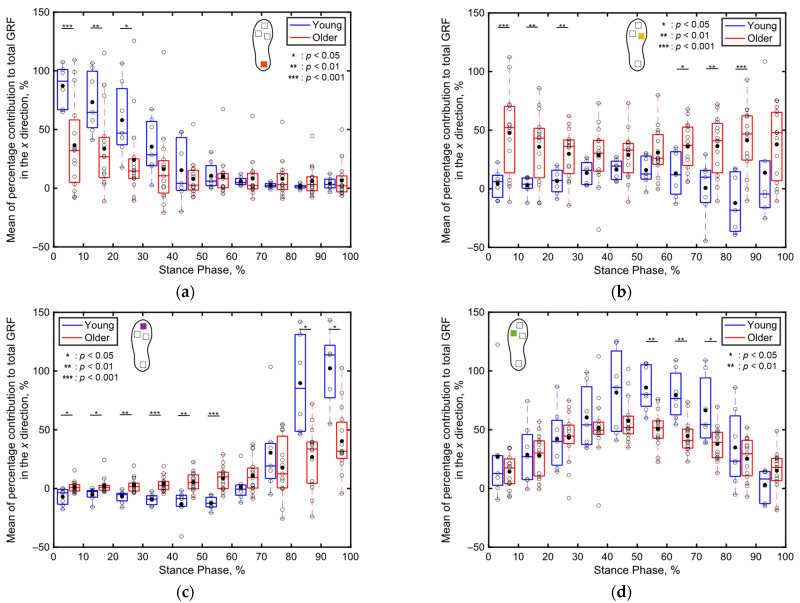
Boxplots depicting the percentage contributions of localized GRFs along the *x* direction at individual sensor locations for the older and young adult participants during the stance phase, divided into 10% increments: (**a**) heel, (**b**) first metatarsal, (**c**) toe, and (**d**) fifth metatarsal. Unfilled markers represent the average values for each participant, while black markers denote the mean values for each age group. *, **, and *** indicate *p* < 0.05, *p* < 0.01, and *p* < 0.001, respectively.

Figure 9 displays the mean percentage contributions of the localized GRFs of both the older and young adult participants along the *x* direction for every 10% of the stance phase at each sensor position, as recorded by the shoe sensor system. In both age groups, the contribution of the heel GRF to the total GRF is greater in the early stance phase (Figure 9a), that of the toe GRF to the total GRF is greater in the late stance phase (Figure 9c), and that of the fifth metatarsal GRF to the total GRF is greater in the middle stance phase (Figure 9d). In contrast, the contribution of the first metatarsal GRF to the total GRF remains minimal throughout the stance phase (Figure 9b).

When focusing on the differences between the age groups, the contribution of the heel’s GRF to the total GRF during 1–30% of the stance phase is significantly lower (*p* < 0.05, Cohen’s *d* > 0.8) for the older adults compared to that for the young adults, as depicted in Figure 9a. Conversely, the contribution of the first metatarsal’s GRF to the total GRF during 1–30% of the stance phase is significantly higher (*p* < 0.01, Cohen’s *d* > 0.8) for the older adults compared to that for the young adults, as illustrated in Figure 9b. Additionally, the contribution of the toe’s GRF to the total GRF along the *x* direction during the first 60% of the stance phase is significantly greater for the older adults (*p* < 0.05, Cohen’s *d* > 0.8) compared to that for the young adults, as depicted in Figure 9c. In the latter half of the stance phase, the contribution of the first metatarsal’s GRF to the total GRF between 61% and 90% of the stance phase is significantly greater (*p* < 0.01, Cohen’s *d* > 0.8) for the older adults compared to that for the young adults, as illustrated in Figure 8b. Meanwhile, the contribution of the toe’s GRF to the total GRF between 81% and 100% of the stance phase and that of the fifth metatarsal’s GRF to the total GRF between 61% and 90% of the stance phase are significantly lower (*p* < 0.05, Cohen’s *d* > 0.8) for the older adults compared to those for the young adults, as illustrated in Figure 9c,d. No significant differences in the localized GRF contributions along the *x* direction are observed among the two age groups in the other intervals of the stance phase.

**Figure 10:**
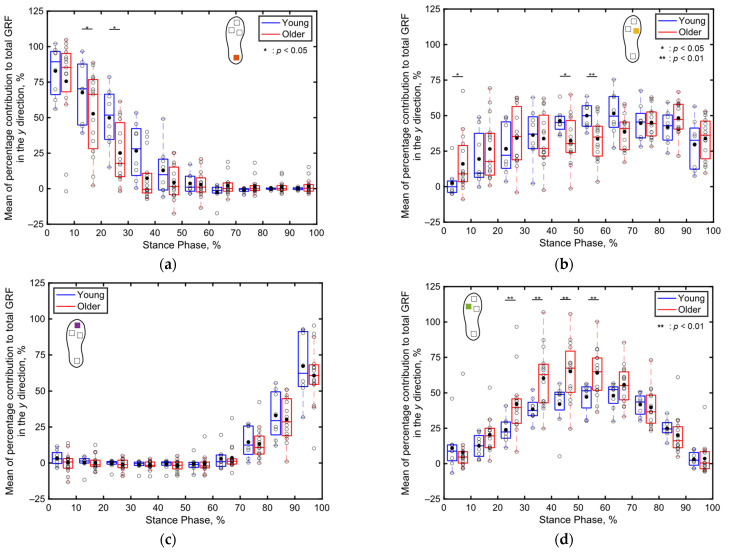
Boxplots depicting the percentage contributions of the localized GRFs along the *y* direction recorded at individual sensor locations for the young and older participants during the stance phase, divided into 10% increments: (**a**) heel, (**b**) first metatarsal, (**c**) toe, and (**d**) fifth metatarsal. Unfilled markers represent the mean values for each participant, while black markers denote the average values for each age group. * and ** imply *p* < 0.05 and *p* < 0.01, respectively.

Figure 10 presents boxplots depicting the percentage contributions of the localized GRFs along the *y* direction recorded at individual sensor positions for both the older and young adult participants. In both age groups, the contribution of the heel GRF to the total GRF is greater in the early stance phase (Figure 10a), that of the toe GRF to the total GRF is greater in the late stance phase (Figure 10c), and that of the fifth metatarsal GRF to the total GRF is greater in the middle stance phase (Figure 10d). In contrast, the first metatarsal GRF primarily contributes immediately after foot contact (Figure 10b).

When focusing on the differences between the age groups, the contribution of the heel’s GRF to the total GRF between 11% and 30% of the stance phase and that of the first metatarsal’s GRF to the total GRF between 41% and 60% of the stance phase are significantly lower (*p* < 0.05, Cohen’s *d* > 0.8) for the older adults compared to those for the young adults, as illustrated in Figure 10a,b. Furthermore, the contribution of the fifth metatarsal’s GRF to the total GRF between 21% and 60% of the stance phase is significantly higher (*p* < 0.01, Cohen’s *d* > 0.8) for the older adults compared to that for the young adults, as depicted in Figure 10d. No significant differences are apparent in the contributions of the localized GRFs along the *y* direction between both age groups in the other intervals of the stance phase.

**Figure 11:**
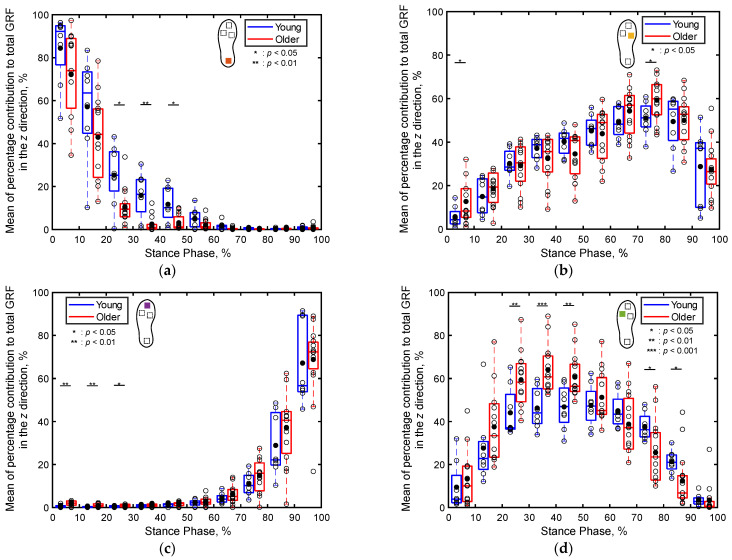
Boxplots depicting the percentage contributions of the localized GRFs along the *z* direction at individual sensor positions for the young and older adult participants during the stance phase, divided into 10% increments: (**a**) heel, (**b**) first metatarsal, (**c**) toe, and (**d**) fifth metatarsal. Unfilled markers denote mean values for each participant, while black markers represent the average values for each age group. *, **, and *** imply *p* < 0.05, *p* < 0.01, and *p* < 0.001, respectively.

Figure 11 presents boxplots depicting the percentage contributions of the localized GRFs along the *z* direction at individual sensor positions for both the older and young adult participants, recorded for every 10% of the stance phase. In both age groups, the contribution of the heel GRF to the total GRF is greater in the early stance phase (Figure 11a), that of the first metatarsal GRF to the total GRF is greater in the late stance phase (Figure 10b), that of the toe GRF to the total GRF is greater in the late stance phase (Figure 11c), and that of the fifth metatarsal GRF to the total GRF is greater in the middle stance phase (Figure 11d).

Regarding the differences between the age groups, the contribution of the heel’s GRF to the total GRF during 21–50% of the stance phase is significantly higher for the young adults compared to that for the older adults (*p* < 0.05, Cohen’s *d* > 0.8), as illustrated in Figure 11a. Conversely, the contribution of the toe’s GRF to the total GRF during 1–30% of the stance phase is significantly higher for the older adults compared to that for the young adults (*p* < 0.05, Cohen’s *d* > 0.8), as depicted in Figure 11c. Furthermore, the contribution of the fifth metatarsal’s GRF to the total GRF during 21–50% is significantly higher for the older adults compared to that for the young adults (*p* < 0.01, Cohen’s *d* > 0.8), as depicted in Figure 11d. In other intervals of the stance phase, no significant differences are apparent in the contributions of the localized GRFs along the *z* direction between the two age groups.

#### 3.2.3. Stride Length

**Figure 12:**
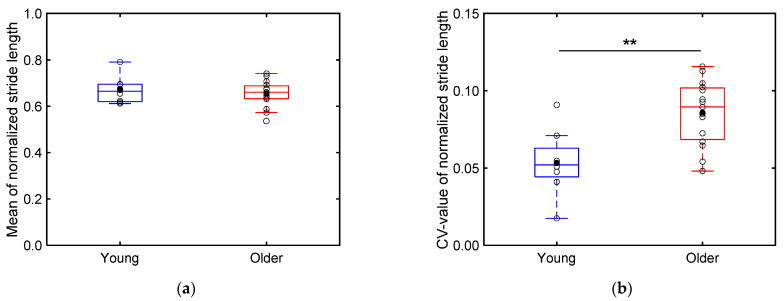
Boxplots depicting (**a**) the normalized stride lengths of the older and young adults and (**b**) the CV values of these normalized stride lengths. Unfilled markers represent the mean value for each participant, while black markers represent the mean value for each age group. ** implies *p* < 0.01.

Figure 12 presents boxplots of the mean (Figure 12a) and CV values (Figure 12b) of the stride length of each participant normalized by their height. As illustrated in Figure 12a, no significant differences are apparent in the mean normalized stride length between the older (0.655 ± 0.057) and young adults (0.670 ± 0.059) (*p* > 0.05). However, as illustrated in Figure 12b, the CV values of the normalized stride length are significantly higher for the older adults (0.0858 ± 0.021) compared to those for the young adults (0.0534 ± 0.021) (*p* < 0.01; Cohen’s *d* > 0.8).

#### 3.2.4. Minimum Toe Clearance

**Figure 13:**
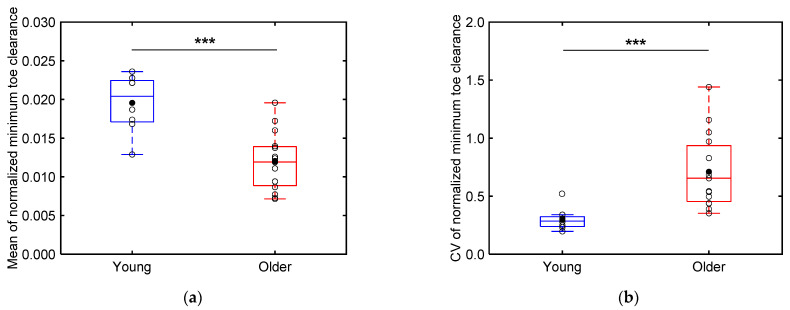
(**a**) Boxplots depicting the normalized minimum toe clearances of the older and young adults and (**b**) the CV values of these normalized minimum toe clearances. Unfilled markers represent the mean value for each participant, while black markers represent the mean value for each age group. *** implies *p* < 0.001.

Figure 13 illustrates boxplots of the mean (Figure 13a) and CV values (Figure 13b) of the minimum toe clearance of each participant normalized by their height. As depicted in Figure 13a, the mean normalized minimum toe clearances are significantly smaller (*p* < 0.001; Cohen’s *d* > 0.8) for the older adults (0.0120 ± 0.0036) compared to those for the young adults (0.0196 ± 0.0035). Furthermore, as illustrated in Figure 13b, the CV values of the minimum toe clearance are significantly higher (*p* < 0.001; Cohen’s *d* > 0.8) for the older adults (0.71 ± 0.31) compared to those for the young adults (0.30 ± 0.09).

## 4. Discussion

We developed a shoe-based sensor system equipped with an IMU and high-capacity, compact triaxial force sensors to simultaneously measure and estimate local GRF distributions in three directions, along with stride length and foot clearance. The results indicate that the developed sensor system provided relatively accurate measurements for stride length and minimum toe clearance compared with conventional optical motion analysis systems. The present study also examined the gait characteristics of young and older adults using our system to confirm previously reported age-related changes and to explore differences in triaxial local GRF distribution between the two groups, which have not been previously documented. The results show that older adults tended to exhibit lower localized three-directional GRF contributions from the heel and higher contributions from the toe and fifth metatarsal locations. Furthermore, the older adults exhibited greater variability in stride length and smaller, more variable minimum toe clearances than the young adults.

### 4.1. Estimation Accuracy of the Stride Length and Minimum Toe Clearance (S1)

Benoussaad et al. [42] introduced a foot clearance estimation approach using an IMU attached to the ankle, aiming to achieve a foot clearance estimation error of less than 0.02 m (minimum foot clearance [3]), in accordance with clinical practice requirements [59]. Remarkably, their method achieved an error of 0.0074 m at normal walking pace [42]. Similar studies have also used IMUs for estimating minimum foot clearance, with Mariani et al. [60] achieving an estimation accuracy of 0.013 m using an IMU on the instep, and Huang et al. [61] reporting an accuracy of 0.0086 m with an IMU mounted on the midfoot of the insole. Notably, using the same estimation algorithm [42] in this study, we obtained an even better estimation accuracy of 0.0056 m, likely owing to the placement of the IMU on the toe. This suggests that our estimation accuracy of foot clearance is adequately high for practical applications. However, the RMSE of stride length in this study is 0.10 m, slightly larger than the estimation errors of 0.04–0.07 m reported in studies estimating stride length by performing second-order integration of acceleration [40,41,47]. Notably, the location of the IMU in these studies was either at the instep, the midfoot of the insole, or at the lateral aspect, right below the ankle joint. The position of the IMU is known to influence the accuracy of stride length estimation performed through the double integral of acceleration [62]. Hence, it is plausible that the placement of the IMU on the toe may have influenced the accuracy of the stride length estimation in this study.

### 4.2. Differences in Localized GRFs Between the Young and Older Adults (S2)

Compared to the three-dimensional total GRF (Figure 8), more significant differences were observed in the localized GRFs among the young and older adults (Figure 9, Figure 10 and Figure 11). Notably, significant age-related differences were evident in the localized GRFs along the *x* direction (Figure 9). For instance, during the 1–60% of the stance phase, the contribution of the heel’s GRF was significantly lower for the older adults compared to that for the young adults (Figure 9a); conversely, the contribution of the first metatarsal’s GRF was significantly higher for the older adults compared to that for the young adults (Figure 9b). In the later stance phase (61–100%), the contribution of the toe’s GRF to the total GRF along the *x* direction was significantly lower among the older adults (Figure 9c). A similar trend was observed along the *y* direction (Figure 10). Along the vertical direction, in the early stance phase, the older adults demonstrated a significantly lower contribution from the heel but considerably greater contributions from the first metatarsal, toe, and fifth metatarsal (Figure 11). These results indicate that the older adults tended to establish foot contact in a flatter orientation owing to their low foot strike angle, demonstrating reduced contribution from the heel and increased contributions from the toe and first metatarsal in the early stance phase. This finding aligns with the results of previous studies, indicating that aging is associated with decreased hip joint angles, a narrow range of motion, increased knee joint angles at foot strike, and decreased extension angles of the hip and knee joints, all contributing to a lower foot strike angle [63,64,65,66,67]. The results of this study support these findings and underscore the unique capabilities of the proposed system to measure localized GRFs, which cannot be measured using force plate systems.

### 4.3. Difference in Stride Length and Minimum Toe Clearance Between the Young and Older Adults (S2)

Our results reveal that the older adult participants exhibited significantly lower mean and considerably larger CV values of the minimum toe clearance compared to the young adult participants (Figure 13). Notably, while the minimum foot clearance does not typically decrease solely owing to aging—often remaining the same or even increasing slightly [3]—elderly individuals are more prone to tripping owing to lower median values of the minimum toe clearance (although not significantly lower) and significantly higher variability in these values [6]. Furthermore, the older adult participants exhibited significantly higher CV values of the stride length compared to the young adults (Figure 12b). This increased variability in both the stride length and toe clearance of older adults has been linked to a higher risk of falls [5,6]. These findings suggest that our shoe sensor system can effectively capture gait differences between young and older individuals, thus presenting an alternative to conventional motion capture systems.

### 4.4. Study Limitation

Despite its contributions, certain limitations of this study must be acknowledged. First, the sample size was small, and the older adult participants were exclusively female, which may limit the generalizability of our results to a broader population. Second, the experimental studies were performed only on one type of flooring. Hence, future research must consider performing such experimental studies on different indoor floorings and outdoor road surfaces to assess the general applicability of the findings. Third, this study solely focused on straight-line walking. Hence, future studies must examine more complex movements, such as turning, which are relevant to daily activities. In addition, slight misalignments between the IMU and force sensors, as well as between individual force sensors, may introduce errors in the GRF and kinematics data, potentially impacting measurement and estimation accuracy.

## 5. Conclusions

This study successfully developed a novel shoe sensor system capable of simultaneously measuring localized GRFs, stride length, and toe clearance during walking. Compared to conventional optical motion analysis systems, our shoe sensor system provided relatively accurate results for both the stride length and minimum toe clearance. Gait experiments conducted using the shoe sensor system revealed that older adults presented lower contribution of the local three-directional GRF from the heel and greater localized GRF contributions from the toe and fifth metatarsal locations, compared to young adults. Furthermore, the older adults exhibited lower minimum toe clearances with greater variability compared to the young adults. The older adults also exhibited greater variability in stride lengths. These findings align with the reports of previous studies and underscore the effectiveness of the developed shoe sensor system in analyzing kinematic and kinetic parameters outside the laboratory environment.

In addition to enabling continuous monitoring of these parameters in real environments, the developed system also enhances our understanding of the complex interactions between biomechanical factors that are otherwise quantifiable only under controlled laboratory settings. By integrating sensors into a pair of shoes, the developed system provides comprehensive data on the mechanical forces and movements involved in daily activities. This advancement offers valuable insights into the biomechanical challenges encountered by different populations, such as the elderly. Furthermore, by correlating changes in gait patterns with GRF data, this approach allows for a more nuanced analysis of walking dynamics, potentially leading to improved designs of assistive devices and targeted interventions to correct gait abnormalities.

## Data Availability

The data, including graphs, within this paper are available from the corresponding author upon reasonable request.
